# Schistosomiasis infection among primary school students in a war zone, Southern Kordofan State, Sudan: a cross-sectional study

**DOI:** 10.1186/1471-2458-13-643

**Published:** 2013-07-11

**Authors:** Alaa H Abou-Zeid, Tigani A Abkar, Rashid O Mohamed

**Affiliations:** 1 Public Health Department, Faculty of Medicine, Cairo University, Kasr Al Ainy St., Cairo, Egypt; 2 State Ministry of Health, Kadugli, South Kordofan, Sudan

**Keywords:** Prevalence, Schistosomiasis, School, Southern Kordofan, Sudan, Survey

## Abstract

**Background:**

Schistosomiasis is a major health problem adversely affecting the health of vulnerable populations in Sudan.

**Methods:**

We conducted a school-based survey to estimate the prevalence of schistosomiasis in 36 villages in Southern Kordofan (SK) State. A total of 2,302 primary school students were recruited. Each student completed a questionnaire and submitted one urine and one stool sample.

**Results:**

The prevalence of schistosomiasis *haematobium* was 23.7%, while schistosomiasis *mansoni* was not detected among the study participants. *S. haematobium* infection was identified in all areas, with the highest prevalence in the western locality of SK State. The infection was associated with the distance between home/school and open water sources. In addition, *S. haematobium* infection was associated with the existence of and distance to open water sources, higher frequency of contact with open water, absence of a health advocacy group in the school and history of schistosomiasis treatment.

**Conclusions:**

This study highlights schistosomiasis as a public health problem in SK State. The findings will guide the schistosomiasis Control Program of the State Ministry of Health in developing and applying treatment plans for schistosomiasis in SK State.

## Background

Schistosomiasis is one of the most prevalent parasitic diseases in the world, with more than 200 million individuals infected at any given time, of whom over half suffer from related morbidity [1,2]. *S. haematobium* and *S. mansoni* are the two species endemic in Sudan. The global burden of schistosomiasis has been estimated at 1.7–4.5 million disability-adjusted life years lost per annum [3], but new research suggests that this estimate is a considerable underestimation of the true burden of schistosomiasis [4-6].

Sudan was the largest country in Africa by land area until the separation of South Sudan in 2011. The ongoing and protracted civil war, recurrent floods, droughts, storms, and a wide range of endemic, epidemic and epizootic diseases constitute important health threats in Sudan [7,8]. Southern Kordofan (SK) State lies on the border between Sudan and South Sudan. For more than 20 years, this state was the scene for war between Sudan and South Sudan that drastically affected the social and economic status of the 1.6 million people living in SK State. Water is provided mainly from dams, wells, superficial rainwater collections and water pumps. Many water ponds are present and stay wet year-round. The availability of latrines in SK State is very low (less than 20%), and the use of available latrines needs to be improved [9].

Schistosomiasis should be identified as a health problem in SK State, particularly among schoolchildren [10]. According to the National Schistosomiasis Control Program [10], the lack of information on schistosomiasis prevalence has stalled the implementation of a treatment strategy. We conducted this study to estimate the prevalence of schistosomiasis infection (*S. haematobium* and *S. mansoni*) and to identify associated risk factors among primary school students in SK State.

## Methods

**Figure 1:**
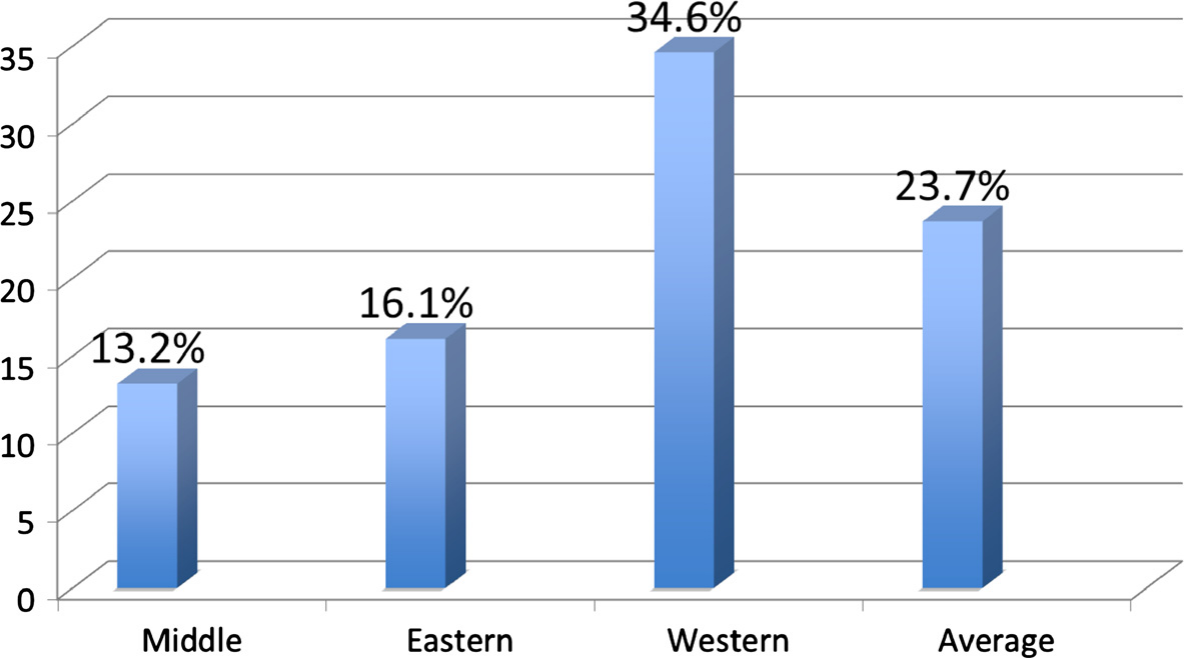
**Prevalence of S. *haematobium *infection among primary schoolchildren by geographic area.**

This was a cross-sectional study to estimate the prevalence of schistosomiasis in South Kordofan State (Figure 1). The study was conducted in all nine localities (the administrative subdivisions of SK State): two central, four western, and three eastern localities.

We used a multistage random sample technique to select students for the study. From each locality, two Administrative Units were randomly selected. Administrative Units are the next smallest subdivision, and each locality contains three to seven Administrative Units. From each Administrative Unit, two villages/towns were randomly selected for the study, for a total of 36 villages/towns. At the village level, the primary school or the nearest primary school was recruited for the study. In each selected primary school, one classroom was selected in each grade from grades 1 to 8. From the selected classroom, seven to eight students were randomly selected and invited to participate in the study by completing a questionnaire, providing at least 10 ml urine, and providing one stool sample.

The only inclusion criterion was that students needed to be registered in the selected primary school. Students who were severely ill at the time of the survey were excluded.

The sample size was estimated using the “World Health Organization Manual for Estimation of Sample Size for Hypothesis Testing” [11]. We estimated the sample size using a 5% sampling error, estimating the prevalence of schistosomiasis at 50%, and with 90% power. This gave a sample size of 1,047, which was doubled to 2,094 to account for study design effect, non-response and missing data.

Each student was interviewed, and data collectors used a structured pre-tested questionnaire that included questions on socioeconomic status of the family and risk factors that may be associated with schistosomiasis infection. Presence of blood in urine was self-reported by students as part of the questionnaire and was compared with the presence of infection as indicated by examination of urine for schistosomiasis eggs. From each recruited student, 10 ml of urine and one stool sample were collected. Stool samples were tested for the presence of *S. mansoni* eggs using the standard Kato Katz technique, while urine was examined using the filtration method [12]. Two slides from the same stool samples were prepared and examined to ensure identification of infection. A second laboratory technician examined 10% of all the slides as a quality control. *S. haematobium* infection was considered severe if egg count was equal or greater than 50 eggs/10 ml of urine. The data collectors also completed a school form that contained data about environmental conditions at the school and in the close vicinity to the school that may pose a risk for schistosomiasis infection. The school data were linked to students’ data and were used to identify risk factors for infection.

Data were entered into a specially designed Microsoft Access 2003 database. The database was converted to Software Package for Social Sciences (SPSS) software version 14.0 for statistical analysis. Age was converted into a categorical variable using suitable cut-off points according to the risk of exposure to polluted water. In this study, children under 8 years of age were assumed to have the lowest risk of exposure, 8–12 year olds were assumed to have a moderate risk of exposure, and children over 12 years of age were assumed to have the highest risk (because of older children being fond of water for recreational purposes). The prevalence and 95% confidence interval (95% CI) of *S. haematobium* infection were computed. Bivariate analysis for the relationship between the prevalence of *S. haematobium* infection and various risk factors was conducted using Pearson Chi square test. Crude odds ratios (ORs) with a 95% CI were calculated and reported for the associations between the prevalence of *S. haematobium* infection and risk factors. The Pearson correlation coefficient was estimated for the correlation between age and ova density in urine. The p value for the Chi Square test was used to build models for multivariate analysis where only variables with p < 0.02 were included. A logistic regression model with backward elimination was developed, and variables that were associated with *S. haematobium* at p < 0.05 were retained in the model.

Based on the multistage design of the study, weighting, stratification, and clustering were taken into account in all statistical analyses using complex samples analysis in SPSS to estimate sampling errors of estimators. This procedure estimates the variance from the variation among the schools, and pools stratum variance estimates to compute the overall variance estimate. A weighting factor was used in the analysis to reflect the likelihood of sampling each student in a stratum. The weight used for estimation is given by the following formula:

W = W1 × W2 × W3; where W1 = the inverse of the probability of selecting the administrative unit in a strata, W2 = the inverse of the probability of selecting the school in a stratum, and W3 = the inverse of the probability of selecting a child from the classroom within the school.

This study was conducted using protocols and tools approved by the Sudanese Federal Ministry of Health. Ethical approval was obtained from the SK State Ministry of Health. Approvals of traditional community leaders were obtained before conducting the survey in any village/town. The survey was explained to schoolmasters and teachers, and their approvals were obtained. Students were informed of the study procedures before participating, and a consent form was given to each student for their guardian’s approval. Student assent was also obtained. Study documents were kept protected in a locked cupboard in the SK State Ministry of Health.

## Results

In this survey 2,302 students were recruited from all nine localities in SK State. All students submitted urine samples and 90.0% of students submitted stool samples. The mean age of students was 10.2 ± 2.5 years and 68.4% were males, which is similar to the ratio of male/female enrollment in schools in the state. Students were primarily recruited from rural areas (81.1%). Illiteracy was 42.5% for fathers of students and 64.6% for mothers of students. The most common occupation of fathers was farming (47.3%), and mothers were mainly housewives (63.7%). More than three-quarters of students (78.5%) mentioned the type of their houses as huts made of local materials (Table 1).

**Table 1:** Sociodemographic characteristics of the study participants, SK State, Sudan

**Variable**		**No (n = 2,302)**	**%**
Location	*Urban*	436	18.9
	*Rural*	1866	81.1
Type of school (n = 36)	*Boys*	9	25.0
	*Girls*	2	5.6
	*Mixed*	25	69.4
Grade	*1st*	328	14.2
	*2nd*	337	14.6
	*3rd*	332	14.4
	*4th*	300	13.0
	*5th*	310	13.5
	*6th*	235	10.2
	*7th*	257	11.2
	*8th*	203	8.8
Gender	*Female*	727	31.6
	*Male*	1575	68.4
Age	*(Mean ± SD)*	10.2	±2.5
Father’s education	*Illiterate*	979	42.5
	*Primary*	997	43.3
	*Intermediate*	137	6.0
	*Secondary*	128	5.6
	*University*	61	2.6
Mother’s education	*Illiterate*	1,487	64.6
	*Primary*	653	28.4
	*Intermediate*	69	3.0
	*Secondary*	69	3.0
	*University*	24	1.0
Father’s occupation	*Farmer*	1,089	47.3
	*Pasturer*	142	6.2
	*Manual worker*	429	18.6
	*Trader*	283	12.3
	*Gov employee*	190	8.3
	*Military*	169	7.3
Mother’s occupation	*Housewife*	1,467	63.7
	*Farmer*	632	27.5
	*Manual worker*	81	3.5
	*Trader*	37	1.6
	*Gov employee*	74	3.2
	*Others*	11	0.5
Type of house	*Fixed*	495	21.5
	*Huts*	1,807	78.5

The prevalence of *S. haematobium* eggs was 23.7% (95% CI 22.0–25.4). The overall ova density among the infected students was 14.9 ± 13.2 eggs/10 ml urine (Table 2). Students with severe infection (≥ 50 eggs/10 ml urine) constituted 4.4% of infected students and 1.0% of the total student population. All students tested negative for *S. mansoni* infection (Table 2).

**Table 2:** Prevalence and severity of schistosomiasis infection among study participants

**Variable**		**Students**	
		**No (n = 2,302)**	**%**
*S. haematobium* infection	*Positive*	545	23.7
Severity of *S. haematobium* infection			
*Mild (eggs 1-25/10 ml urine)*		364	15.8
*Moderate (eggs 26-49/10 ml urine)*		172	7.5
*Severe (eggs* ≥*50/10 ml urine)*		9	0.4
		*Mean*	*±SD*
Severity of *S. haematobium* infection	*(eggs/10 ml urine)*	14.9	*±*13.2
*S. mansoni* infection*	*Positive*	0/2,072	0.0%

*Not all subjects submitted stool samples.

**Figure 2:**
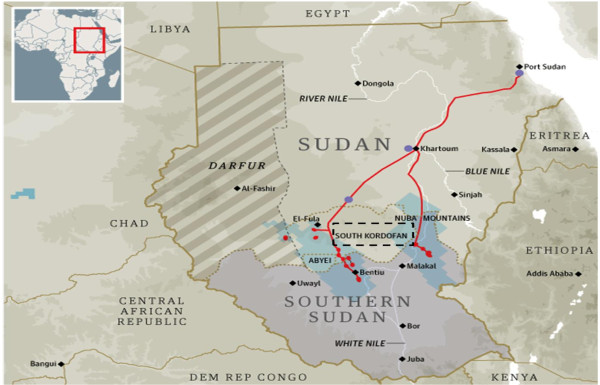
Location of the study area.

The prevalence of *S. haematobium* differed by locality, administrative unit and school, with a range of 0–80% per school. The highest prevalence was reported in the western area of the state (34.6%), followed by the eastern (16.1%) and then the central area (13.2%) (Figure 2). The students were divided into three age groups to estimate the association between age and *S. haematobium* infection (Table 3). While the prevalence of infection was inversely proportional to age, this relationship was not significant (p value = 0.34). Infection was slightly higher among males (24.8%) than females (21.2%). Students with illiterate fathers had a higher prevalence of infection (p value =0.04). Similarly, maternal illiteracy was also associated with infection (p value =0.006). Paternal and maternal occupations were each categorized into two levels based on the types of work that necessitates contact with open water sources. All paternal work in Sudan was outdoors; maternal work as a farmer (outdoor occupation) was significantly associated with infection among children (p value =0.035). The school environment was strongly associated with *S. haematobium* infection among students. Schools with an open water source less than 1 km away, absence of hand-washing facilities at schools, and absence of a closed water source (such as piped water) at school were significantly associated with infection (p value <0.001). In addition, infection was higher among students who had frequent contact with open water sources (more than once a week), students who did not have information about the disease and students who had previously received treatment for schistosomiasis (p value <0.001). The presence of health advocacy groups at school and the presence of a health facility in the same village were associated with less infection among students (p value <0.001) (Table 3). There was no correlation between ova density and age (r = 0.028 and p value = 0.52). Similarly, no difference was detected in mean ova density between males (mean = 15.1) and females (mean = 14.9; p value = 0.88).

**Table 3:** **General demographic characteristics, crude and adjusted odds ratios of *S. haematobium *by risk factors**

**Variable**		***S. haematobium *infected**	**Crude**	**Adjusted**
		**No (%)**	**OR (95% CI)**	**OR (95% CI)**
Location	*Urban*	36/437 (8.2%)	1	1
	*Rural*	509/1866 (27.3%)	4.17 (2.92-5.95)	4.70 (3.13-7.08)
Geographic part within the State	*Central*	68/514 (13.2%)	1	1
	*Eastern*	124/768 (16.1%)	1.26 (0.92-1.74)	1.02 (0.69-1.49)
	*Western*	353/1020 (34.6%)	3.47 (2.61-4.62)	1.82 (1.27-2.59)
Age	*>12 ys*	101/460 (22.0%)	1	Removed
	*8-12 ys*	274/1177 (23.3%)	1.08 (0.83-1.40)	
	*<8 ys*	170/665 (25.6%)	1.22 (0.92-1.62)	
Gender	*Female*	154/727 (21.2%)	1	1
	*Male*	391/1575 (24.8%)	1.23 (0.99-1.52)	1.25 (0.97-1.60)
Father’s education	*Above primary*	56/308 (18.2%)	1	Removed
	*Primary*	229/963 (23.8%)	1.40 (1.01-1.94)	
	*Illiterate*	260/1031 (25.2%)	1.52 (1.10-2.09)	
Mother’s education	*Above primary*	24/153 (15.7%)	1	Removed
	*Primary*	139/653 (21.3%)	1.45 (0.91-2.34)	
	*Illiterate*	382/1496 (25.5%)	1.84 (1.17-2.89)	
Father’s Occupation	*Indoor worker*	240/1071 (22.4%)	1	1
	*Outdoor worker*	305/1231 (24.8%)	1.14 (0.94-1.38)	0.80 (0.64-1.00)
Mother’s Occupation	*Indoor worker*	36/203 (17.7%)	1	Removed
	*Housewife*	342/1467 (23.3%)	1.41 (0.96-2.10)	
	*Farmer*	167/632 (26.4%)	1.67 (1.12-2.49)	
Type of house	*Fixed*	103/495 (20.8%)	1	Removed
	*Cottage*	442/1807 (24.5%)	1.23 (0.97-1.57)	
Source of water	Closed *	284/1579 (18.0%)	1	1
	*Opened*	261/723 (36.1%)	2.58 (2.11-3.14)	2.96 (2.34-3.74)
Hand washing facility at school	*Available*	105/606 (17.3%)	1	1
	*Not available*	440/1696 (25.9%)	1.67 (1.32-2.12)	1.51 (1.15-1.99)
Latrine at school	*Available*	247/1062 (23.3%)	1	Not included
	*Not available*	298/1240 (24.0%)	1.04 (0.86-1.27)	
Distance from school to nearest open water source	*≥ 1 Km*	9/142 (6.3%)	1	32.50 (12.89-81.95)
	*< 1 Km*	536/2160 (24.8%)	4.88 (2.47-9.65)	
Play/bath in open water	*No*	152/833 (18.2%)	1	1
	*Yes*	393/1469 (26.8%)	1.64 (1.33-2.02)	1.48 (1.15-1.89)
Frequency of open water contact	*>weekly*	306/1660 (18.4%)	1	1
	*Daily*	239/642 (37.2%)	2.62 (2.14-3.21)	3.92 (3.06-5.02)
Health advocacy at school	*Yes*	115/642 (17.9%)	1	Removed
	*No*	430/1660 (%)	1.60 (1.27-2.02)	
Health facility in the same village	*Yes*	277/1349 (20.5%)	1	1
	*No*	268/953 (28.1%)	1.51 (1.23-1.84)	1.53 (1.21-1.93)
Knowledge about schistosomiasis	*Yes*	210/941 (21.8%)	1	1
	*No*	335/1361 (26.7%)	1.45 (1.19-1.76)	1.48 (1.18-1.86)
History of schistosomiasis treatment	*No*	462/2123 (21.8%)	1	1
	*Yes*	83/179 (46.4%)	3.11 (2.28-4.24)	3.37 (2.53-4.51)

* *Pipe/hand pump.*

Multivariate analysis showed that living in a rural area carried about five times the risk of infection compared with living in an urban area (OR 4.7; 95% CI 3.13–7.08). Living in the western locality carried twice the risk of infection compared with the central area of SK State (OR 1.82; 95% CI 1.27–2.59). Using an open water source carried almost three times the risk of infection (OR 2.96; 95% CI 2.34 –3.74), lack of hand-washing facilities at school carried 1.5 times the risk (OR1.51; 95% CI 1.15–1.99) and distance less than 1 km between an open water source and the school led to over 32 times the infection risk of the comparison group (OR 32.5; 95% CI 12.89–81.95).

Other factors that were associated with infection were swimming and bathing in open water sources (OR1.48; 95% CI 1.15-1.89), contact with open water sources for more than once a week (OR 3.92; CI 3.06-5.02) compared with contact for less than once a week, absence of a health facility at the same location (Or 1.53; CI 1.21-1.93), student being unaware of schistosomiasis (OR 1.48; CI 1.18-1.86) and history of receiving schistosomiasis treatment (OR 3.37; CI 2.53-4.51) (Table 3).

Self-report of passing red urine was insensitive as a predictor of *S. haematobium* infection among students. The sensitivity of reporting passing red urine was 140/545 (25.7%). The specificity of reporting passing red urine was 89.0% (Table 4). Sensitivity was slightly better in male students (27.1%) compared with females (22.1%). The positive predictive value of reporting passing red urine was 41.9%, and the negative predictive value was 79.5%. The overall accuracy of self-reporting passing red urine was 73.0% (data not shown in tables).

**Table 4:** **Sensitivity and specificity of self-reported passing of red urine as a predictor for *S. haematobium *infection among students**

		**Microscopy for *S. haematobium***		**Total**
		**Positive**	**Negative**	
**Red urine**	+ve	140 (25.7%)	194 (11.0%)	334 (14.5%)
	-ve	405 (74.3%)	1563 (89.0%)	1968 (85.5%)
	**Total**	545 (100.0%)	1757 (100.0%)	2302 (100.0%)

## Discussion

To our knowledge, this is the first State-wide schistosomiasis survey in SK State. The prevalence of *S. haematobium* infection was 23.7% among schoolchildren. *S. mansoni* infection was not detected in SK State among students. The prevalence of *S. haematobium* varied greatly at different locations around the State. The western locality, had significantly higher rates of infection. *S. haematobium* infection was significantly associated with many environmental conditions such as using open sources of water, lacking hand-washing facilities at school and proximity between school and an open water source.

The estimated prevalence of schistosomiasis has varied considerably among different studies, but has been reported to be 40% among schoolchildren in Ghana [13]. The long-running civil war in Sudan has devastated social and health services. Thus, updated epidemiologic data are lacking and some epidemiologic reports are scattered, incomplete, and outdated, and most are unpublished. Old data from 1930–1970 reported that *S. haematobium* affected 2.5% and *S. mansoni* affected 1.9% of the population in parts of Southern Sudan [14]. The estimated prevalence in our study was similar to more recent studies, such as a report that the prevalence of *S. haematobium* was 21.4% and *S. mansoni* was 10.1% in White Nile State [15]. In 2007, Deganello et al. found the prevalence of *S. haematobium* to range from 0–73% among students from primary schools in two regions in Southern Sudan [14]. In Malawi, Atupele et al. reported the prevalence of *S. haematobium* to be 10.4% among primary school students [16]. The estimated prevalence of *S. haematobium* and *S. mansoni* in our study is quite similar to the finding of a recent national schistosomiasis study among students in Malawi, which reported a prevalence of 2.0–23.2% for *S. haematobium* and 0.0–1.3% for *S. mansoni*[17]. *S. haematobium* infection in our study is more prevalent among schoolchildren compared with the adult population in SK State (6.9%), and the absence of *S. mansoni* is consistent with findings among the adult population in SK State [18].

The severity of *S. haematobium* infection varies between studies. Deganello et al. reported 0.0–28.5% of the positive students examined from two regions as having high intensity (≥50 eggs/10 ml urine) [14]. Our estimation for the severity of infection among students in SK State (4.4%) lies within the findings of this study.

We did not find a difference in prevalence between male and female students, which is similar to previous findings [14,15]. However, other studies found higher prevalence rate of *S. haematobium* infection among male students in Malawi [16,19]. The difference in prevalence could be attributed to the fact that people in SK State depend to a larger extent on open water sources for household purposes, with males and females being similarly exposed. The prevalence of *S. haematobium* infection was not associated with the age of students in our study, which was similar to the findings in a White Nile survey [15]. The heavy dependence on open water could also possibly explain the similar prevalence among various age groups in our study.

We found an association between the levels of parental education and *S. haematobium* infection, as well as between certain types of occupation of *S. haematobium* infection. This finding is similar to that reported by others [20,21], but conflicting results have been reported from Malawi [16]. The proximity of open water sources has been consistently reported as associated with *S. haematobium* infection [22,23], and we also found this association.

A history of urinary schistosomiasis treatment was strongly associated with *S. haematobium* infection in SK State, which is similar to the findings of Atupele et al. in Malawi [16]. This could be explained by the fact that communities with high prevalence rates tend to cluster around contaminated water sources even after being treated for schistosomiasis [24].

The specificity of self-reporting of passing red urine in our study was consistent with the specificity reported from other studies, which range from 58–96% [25,26]. However, the sensitivity we reported (25.7%) was less than the average reported in the two previously mentioned studies (50–100%). A possible reason for this is that poor students in Sudan may have been afraid to be referred to the hospital for expensive treatment if they reported passing red urine.

## Conclusion

*S. haematobium* is a significant problem adversely affecting the health of vulnerable populations in SK State. The burden in schoolchildren is higher compared with the rest of the population. The estimation of the prevalence among schoolchildren can reflect the burden among the rest of the population at the village/town level. Our study showed that *S. mansoni* was not detected among students in SK State. The use of interviews using a questionnaire as a screening tool for *S. haematobium* infection is not valuable because of its low sensitivity compared with urine examination for *S. haematobium* eggs, which is the gold standard test for diagnosing the infection. This study illustrates the national efforts to better control schistosomiasis activities by fostering the Schistosomiasis Control Programs at the State level in Sudan. Repetition of this survey in SK State in school students and in the general population is recommended to update the treatment policy of the State Ministry of Health. Similar State-wide surveys are also recommended to guide treatment in other states. In addition, studies on different treatment schemes that may guide better policies to ensure timely and better use of praziquantel is recommended.

## Competing interests

The authors declare that they have no competing interests.

## Authors’ contributions

AA was responsible for the design in terms of methods, sample size and statistical concerns. He also guided the execution of the study, conducted the statistical analysis and participated in the writing of the paper. TA initiated the idea for this work, participated in and provided information necessary for the design, and guided the design for suitability of the State context. He supervised the execution of the fieldwork and participated in the writing and reviewing of the paper. RM was responsible for the recruitment of the study data collectors and laboratory technicians, and their training, supervision and troubleshooting of the study in the field, including all necessary logistics. He participated in the writing and reviewing of the paper. All authors read and approved the final manuscript.

## Authors’ information

AA is an Associate Professor of Public Health at the Faculty of Medicine, Cairo University. His work focuses on epidemiologic research, especially infectious diseases epidemiology. TA is the director of the State Ministry of Health in Southern Kordofan. His background is internal medicine. RM is the director of the Schistosomiasis Control Program in the State Ministry of Health.

## Pre-publication history

The pre-publication history for this paper can be accessed here:

http://www.biomedcentral.com/1471-2458/13/643/prepub
